# Clinically relevant copy number variations detected in cerebral palsy

**DOI:** 10.1038/ncomms8949

**Published:** 2015-08-03

**Authors:** Maryam Oskoui, Matthew J. Gazzellone, Bhooma Thiruvahindrapuram, Mehdi Zarrei, John Andersen, John Wei, Zhuozhi Wang, Richard F. Wintle, Christian R. Marshall, Ronald D. Cohn, Rosanna Weksberg, Dimitri J. Stavropoulos, Darcy Fehlings, Michael I. Shevell, Stephen W. Scherer

**Affiliations:** 1 Departments of Pediatrics and Neurology/Neurosurgery, McGill University, Montreal, Quebec, Canada H3H 1P3; 2 The Centre for Applied Genomics, The Hospital for Sick Children, Toronto, Ontario, Canada M5G 0A4; 3 Program in Genetics and Genome Biology, The Hospital for Sick Children, Toronto, Ontario, Canada M5G 0A4; 4 Department of Pediatrics, University of Alberta, Edmonton, Alberta Canada T6G 2B7; 5 Glenrose Rehabilitation Hospital, Edmonton, Alberta, Canada T5G 0B7; 6 Genome Diagnostics, Department of Pediatric Laboratory Medicine, The Hospital for Sick Children, Toronto, Ontario, Canada M5G 1X8; 7 Department of Paediatrics, University of Toronto, Toronto, Ontario, Canada M5G 1X8; 8 Centre for Genetic Medicine, The Hospital for Sick Children, Toronto, Ontario, Canada M5G 1X8; 9 Division of Clinical and Metabolic Genetics, The Hospital for Sick Children, Toronto, Ontario, Canada M5G 1X8; 10 Institute of Medical Science, University of Toronto, Toronto, Ontario, Canada M5G 1X8; 11 Department of Laboratory Medicine and Pathobiology, The Hospital for Sick Children, Toronto, Ontario, Canada M5G 1X8; 12 Holland Bloorview Kids Rehabilitation Hospital, Department of Paediatrics, University of Toronto, Toronto, Ontario, Canada M4G 1R8; 13 Department of Molecular Genetics and McLaughlin Centre, University of Toronto, Toronto, Ontario, Canada M5S 1A8

## Abstract

Cerebral palsy (CP) represents a group of non-progressive clinically heterogeneous disorders that are characterized by motor impairment and early age of onset, frequently accompanied by co-morbidities. The cause of CP has historically been attributed to environmental stressors resulting in brain damage. While genetic risk factors are also implicated, guidelines for diagnostic assessment of CP do not recommend for routine genetic testing. Given numerous reports of aetiologic copy number variations (CNVs) in other neurodevelopmental disorders, we used microarrays to genotype a population-based prospective cohort of children with CP and their parents. Here we identify *de novo* CNVs in 8/115 (7.0%) CP patients (∼1% rate in controls). In four children, large chromosomal abnormalities deemed likely pathogenic were found, and they were significantly more likely to have severe neuromotor impairments than those CP subjects without such alterations. Overall, the CNV data would have impacted our diagnosis or classification of CP in 11/115 (9.6%) families.

## Abstract (web summary)

Cerebral palsy (CP) is a heterogeneous disorder that has been historically attributed to environmental factors with genetic contributions being discovered more recently. Here the authors perform microarray-based analysis of copy number variations in a cohort of children with CP and their parents and find chromosomal abnormalities linked to the disease.

## Graphical abstract

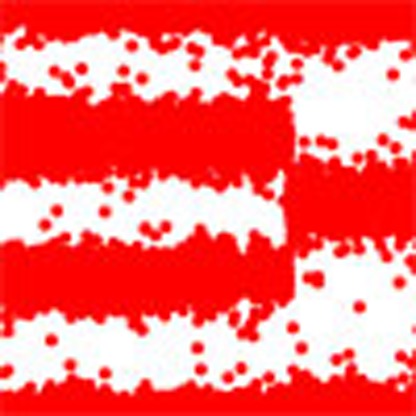

Cerebral palsy (CP) is the most common cause of childhood physical disability, affecting ∼2.11 per 1,000 live births in high-resource settings^1^. CP arises from a non-progressive pathology that affects the developing brain either pre-, peri- or postnatally. Some of the established risk factors for CP include prematurity, intra-uterine growth restriction (IUGR), intra-uterine infections, birth defects and neonatal encephalopathy of various causes including perinatal asphyxia^2^,^3^,^4^. Recent studies suggest that birth asphyxia explains <10% of cases of neonatal encephalopathy^5^. Furthermore, there is tremendous variability in outcomes for any given risk factor, suggesting that some children may have an inherent higher susceptibility to CP. The consensus definition of CP^6^ includes associated co-morbidities, such as epilepsy, communication impairment, sensory impairments or cognitive deficits, which greatly contribute to the associated health burden for children and families.

CP can be classified by neurological subtype as well as by several functional classification systems. The neurological subtype stratification is based on the topographic distribution of affected limbs and the predominant quality of the observed motor impairment, grouping CP into subtypes: spastic quadriplegia, spastic diplegia, spastic hemiplegia, dyskinetic or dystonic, ataxic or hypotonic, or mixed. The motor severity can be described using the Gross Motor Function Classification System (GMFCS), which uses scores that range from most able (level I) to least able (level V)^7^.

Twin and family studies suggest a genetic contribution to some CP^8^,^9^. Consanguineous families carrying recessive mutations in the glutamate decarboxylase 1 (*GAD1*) gene have been described resulting in impaired production of γ-aminobutyric acid^10^. Treatment with drugs that potentiate γ-aminobutyric acid (for example, baclofen and benzodiazepine) ameliorate muscle rigidity and spasticity in these individuals, and have also been more generally used in CP^11^.

Microdeletions of *KANK1* have been identified in nine individuals with quadriplegia and intellectual impairment in a four-generation family^12^. Neuroimaging revealed brain atrophy and ventriculomegaly^13^, consistent with *KANK1*'s role in neuronal signalling and adhesion. Other studies have identified autosomal recessive variants in the adaptor protein complex 4 family (*AP4B1*, *AP4E1*, *AP4M1* and *AP4S1*)^14^,^15^ of molecules involved in neuronal polarity^16^. Gene-based association studies testing for the potential role of common genetic variants in CP have not yet revealed bona fide risk loci^15^.

To date, the literature on the genetic predisposition to CP has focused on selected patient populations with positive family history, dysmorphic features or atypical clinical features such as normal brain imaging. Routine genetic studies are not yet recommended in the diagnostic assessment of children with CP, especially in children where other risk factors are identified^17^. The recognition of the importance of *de novo* and rare inherited copy number variations (CNVs) in the clinical manifestations of a growing list of neurodevelopmental conditions (Table 1) prompted us to test whether genomic abnormalities might similarly be observed in CP^18^. Our initial hypothesis was that *de novo* CNVs would be seen in an unselected cohort of children with CP more frequently than in the general population. Here we identify clinically relevant *de novo* and rare inherited CNVs at a comparatively high frequency that contributes to the aetiology of CP.

## Results

### Clinically relevant variants

Following our rigorous quality control procedure for CNV detection, 147 probands (81 males and 66 females) and 282 parents were used for the subsequent genomic analysis. The clinical characteristics of these children are described in Table 2 and specific cases are described in detail below. From this data, we identified 412 rare CNVs (in <0.1% of our population controls) in the probands (Supplementary Data set 1). We compared the average number of unannotated calls and average CNV size between CP subjects and parents and found no significant difference for either of these measures using an unpaired Student's *t*-test (two-tailed *P* values are 0.22 and 0.49, respectively) (Supplementary Table 1). Among our samples were 115 complete parent–child trios (64 males and 51 female probands) that yielded sufficiently high-quality data allowing assessment of the segregation status of CNVs. Potentially contributory *de novo* or rare inherited CNVs were found in 11/115 families (Table 3) and each of these was validated using the SYBR Green-based real-time quantitative PCR assay^19^. The clinical characteristics of these individuals are summarized in Supplementary Data set 2. The potential clinical impact of CNVs was determined by comparison with the Database of Genomic Variants^20^ and clinical CNV databases^21^.

For those four individuals (4-13C, 10-032C, 10-012C and 13-026C) with large (>5 Mb) CNVs deemed pathogenic or likely pathogenic and discussed in detail below, we tested whether they were more likely to have severe motor impairments. We examined the GMFCS scores provided at the time of registration for 103 of the probands and classified them as having mild-moderate (GMFCS I–III) or severe (GMFCS IV–V) motor impairments. One of the 78 cases with a GMFCS from I–III had a large *de novo* CNV (13-026C), while 3 of 25 with a score of IV or V had a large *de novo* variant. Using a two-tailed Fisher's exact test, we confirmed that individuals with large CNVs were more likely to be severely impaired (*P*=0.04).

### *De novo* CNVs

*De novo* CNVs were identified in 8/115 (7.0%) trios and 4/115 (3.5%) of these subjects carried massive chromosomal structural abnormalities, which were likely the cause of CP, an associated disorder misdiagnosed as CP, and/or other co-morbidities in these individuals. Female 4-13C with dyskinetic CP—choreoathetotic subtype (GMFCS IV) carried a 74 Mb *de novo* duplication (371 genes; 2p25.3-2p13.1) and a 31 Mb *de novo* deletion (165 genes; Xp22.33-Xp21.2), suggestive of an unbalanced translocation. Her birth was induced at 39 weeks and she needed to be resuscitated. Magnetic resonance imaging identified a nonspecific bilateral white matter signal abnormality, bilateral cortical dysplasia in Sylvian areas and hypoplasia of the anterior falx cerebri.

Female 10-032C with spastic quadriplegia (GMFCS V) carried *de novo* hemizygous deletions of 25.5 Mb (134 genes; 4p16.3-4p15.2) and 8.1 Mb (*KANK1* and 38 other genes; 9p24.3-9p24.1). Born at 34 weeks, she was 1.63 kg at birth with a head circumference of 27.5 cm and showed evidence of IUGR. Her mother had previous miscarriages and likely carries a balanced translocation. A cranial computed tomography scan identified improper formation of the subarachnoid space and hypoplasia. She is cognitively impaired, has seizures and severe scoliosis.

Male 10-012C with spastic quadriplegia (GMFCS IV) has a 3.1 Mb *de novo* deletion (9 genes; 2p25.3), an adjacent *de novo* 12.1 Mb duplication (43 genes; 2p25.3-2p24.3), as well as a 43 kb *de novo* microdeletion affecting *RAPGEF1* at 9q34.13. He was born at 40 weeks by spontaneous vaginal delivery, and exhibited motor and developmental problems and an ultrasound identified hydrocephalus. He was non-communicative, had visual impairment and strabismus.

Male subject 13-026C, with dyskinetic CP—choreoathetotic subtype phenotype (GMFCS II)—was found to carry 5.8 Mb *de novo* deletion (18 genes) at 15q11.2-15q13.1 encompassing the type-I deletion associated with Angelman syndrome. The child was non-verbal, had cognitive impairments, and seizures before 25 months, but interestingly was not described as being ataxic. At age 5 years, his diagnosis was changed from CP to Angelman syndrome, as would be predicted by the microarray findings.

A fifth subject, male 6-06C, with right spastic hemiplegia (GFMCS I) had a 2.8 Mb *de novo* duplication (20 genes; 22q13.31) characterized as being likely pathogenic. This CNV is below our 5 Mb cutoff described above and it affects an under-characterized region of the genome, so for now it is not yet deemed pathogenic. However, the large number of genes affected and its *de novo* occurrence place it in the category of having ‘likely clinical consequence'. His mother was hospitalized once during pregnancy for dehydration and gestational thrombocytopenia. The child was born at 39 weeks gestation with signs of neonatal encephalopathy. A computed tomography scan undertaken within 1 week of birth detected an ischaemic lesion in the frontoparietal area (both cortical and subcortical) around the left Sylvian artery.

**Figure 1:**
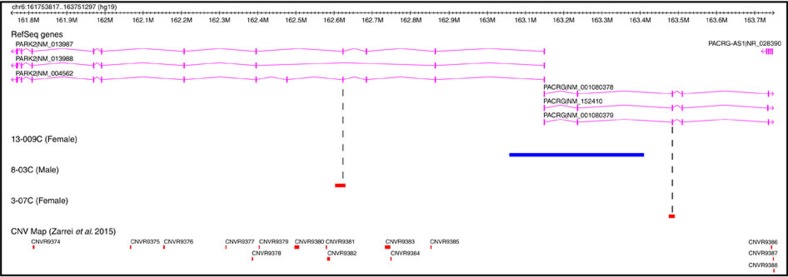
CNVs affecting *PARK2* and *PACRG*. The *de novo* duplication in patient 13-009C impacting both *PARK2* and *PACRG* is illustrated by the blue bar above. Inherited deletions (denoted by the red bars) have been identified in exon 4 of *PARK2* (NM_004562.2) in subject 8-03C and in exon 4 of *PACRG* (NM_152410.2) in subject 3-07C.

The three remaining CP cases carried smaller *de novo* CNVs, each characterized as a Variant of Unknown Significance due to a paucity of published reports regarding duplications of these genes^22^: female 13-009C with spastic quadriplegia (GMFCS V), female 4-10C with spastic diplegia (GMFCS I) and male 13-016C with dyskinetic CP (GMFCS V) had a 351 kb duplication (involving the first exon of *PARK2* and the first two exons of *PACRG*) (Fig. 1 and below), a 48 kb duplication (affecting *HSPA4*) and 29 kb deletion (upstream of *WNT4*), respectively. Female 13-009C had signs suggestive of an acute intrapartum hypoxic event and needed resuscitation at birth. A head ultrasound identified diffuse oedema and slit-like ventricles consistent with hypoxic-ischaemic encephalopathy. Female 4-10C had no apparent prenatal or perinatal risk factors or other co-morbidities, but 13-016C experienced prenatal risk factors for CP and was born by emergency C-section at 24 weeks. A magnetic resonance imaging identified a stage II intraventricular haemorrhage (illustrating bleeding inside the ventricles) and diffuse white matter injury (as expected given prematurity). Without additional genetic findings in unrelated patients, such variants are of uncertain clinical relevance.

### Rare inherited CNVs

Rare inherited CNVs affecting loci of known clinical genetic significance were detected in 3/115 (2.6%) of additional families. CP case 8-02C has spastic diplegia (GMFCS I) and carried a 2.1 Mb maternally inherited microdeletion (18 genes; 1q21.1-1q21.2), which exhibits variable phenotypic expression^23^,^24^. The prenatal and perinatal insults experienced likely caused CP, and the 1q21.1 microdeletion likely contributed to other complications such as IUGR and vision problems. Case 10-027C with spastic right hemiplegia (GMFCS I) carried a 1.4 Mb maternally inherited duplication (15 genes; 16p13.11), which may have contributed to her CP-associated co-morbidities of deficits in communication abilities^25^. Female 10-006C had spastic right hemiplegia (GMFCS II) and a paternally inherited duplication affecting part of the X-linked *DMD* muscular dystrophy gene, which is predicted to be likely benign.

We also identified *de novo* and rare inherited CNVs affecting genes (*PARK2*, *PACRG* and *HSPA4*) involved in the unfolded protein response to endoplasmic reticulum stress, potentially providing insight into genetic and environmental interplay in CP (Fig. 1). Three unrelated patients carried CNVs affecting the *PARK2/PACRG* locus, two overlapping genes co-regulated with a common bidirectional promoter^26^. In addition to the 351 kb duplication affecting the first exon of *PARK2* and the first three exons of *PACRG* in case 13-009C described above, male 8-03C with spastic quadriplegia (GMFCS IV) and female 3-07C with spastic left hemiplegia (GMFCS II) carried a 26 kb deletion of exon 4 in *PARK2* and a 14 kb deletion of exon 4 in *PACRG*, respectively. All three of these subjects experienced some form of pre-, peri- or postnatal insult, which likely led to their CP. Case 4-10C with spastic diplegia (GMFCS I) had a *de novo* 48 kb duplication affecting *HSPA4* (mentioned previously), but no other risk factors for CP.

Mutations in *PARK2* are among the most common causes of autosomal recessive early-onset Parkinson's disease and haploinsufficiency of the gene has also been shown to be a risk factor of familial forms^27^. PARK2 plays an important role in the ubiquitin–proteasome system in the endoplasmic reticulum, a process that targets proteins for destruction. PACRG, the Parkin co-regulated gene product, forms a complex with heat shock proteins (including HSPA4) and other chaperones to suppress cell death in response to an accumulation of unfolded protein. Given our CNV finding affecting these endoplasmic reticulum genes in CP, we speculate that a developmental insult(s) could perhaps elicit an unfolded protein response from prosurvival to the damaging pro-apoptotic phase elevating CP risk. It has been shown that immature neurons and preoligodendrocytes, which contribute to white matter formation, are particularly vulnerable to apoptosis as a result of endoplasmic reticulum stress^28^.

## Discussion

Our genome-wide analysis yields new results indicating that large chromosomal abnormalities can be involved in CP. Prompted by our observations, we found deep in the literature an earlier study that used karyotyping to identify chromosomal anomalies in 8/100 (8%) individuals with CP^29^. The role of these anomalies in the pathogenicity of CP would require further assessment since in 6/8 cases the rearrangements were balanced or inherited, and one had 46, XYY aneuploidy, which is not known to be associated with CP. Most interestingly, the remaining subject had the Angelman syndrome deletion (15q11-q12) similar to 13-026C in our study.

A recent microarray study of 52 CP families ascertained to be cryptogenic (no known aetiology) described 7/52 (13%) subjects as carrying *de novo* CNVs deemed pathogenic (one affecting a locus, *KANK1*, found in our study and others)^30^. In our trio-based analysis, 10/101 individuals for which we had relevant data were cryptogenic, and 1/10 (10%) of these carried a *de novo* potentially clinically relevant CNV. A separate study of 50 unselected CP families failed to detect any *de novo* CNVs, but did find that ∼20% had a rare inherited CNV(s) of potential clinical relevance to the patient^31^. Recent exome sequencing studies of families^32^,^33^ have also identified *de novo* sequence-level variants in CP subjects in some genes (for example, *AGAP1*, *L1CAM, PAK3, TENM1* and *TUBA1A*) with potential functional relevance to the disorder. Two of these CP candidate genes (*AGAP1* and *TENM1*)^33^ found by sequencing are also affected by CNV loci found in our study. As with our findings at the *PARK2* locus (Fig. 1), further replication experiments, however, are required to determine the pathogenicity of these genes before assigning clinical impact in CP.

Taken together, there is a surge of new CNV data, and sequencing results that suggests a genomic basis for CP needs to be considered. In our systematic study, we determined a 7.0% *de novo* CNV rate in a population-based CP cohort, and ∼10% of the families studied carried clinically relevant CNVs that either explain the aetiologic basis of CP or possibly account for associated medical complications. The differences in our findings compared with other studies are likely due to the small samples sizes so far examined and different ascertainment strategies for CP, both being compounded by what appears to be a genetically heterogeneous disorder.

Notwithstanding these complexities, for the majority of families in Table 3, having the genetic data early would have enabled the recognition of a specific aetiology/diagnosis facilitating more accurate management, and counselling regarding recurrence risk. For example, case 13-026C was eventually clinically diagnosed with Angelman syndrome at age 5 years and had microarray analysis been performed earlier, his family would have received more accurate information about the natural history of his diagnosis (there is minimal, if any, speech development in Angelman syndrome). Similarly, in cases 4-13C, 10-032C and 10-012C carrying large CNVs aetiologic for the CP, a more accurate attribution of cause and genetic counselling would have ensued. In our experience, the remaining seven CP cases in Table 3 would also have been seen in clinical genetics and directed to the appropriate specialist.

Importantly, finding another primary diagnosis (for example, microarray-based detection of chromosomal abnormalities and Wolf–Hirschhorn syndrome in 10-032C) does not negate a diagnosis of CP. A complete understanding of the effects of genotypes and environmental stressors on the clinical presentation(s) and manifestations of CP will require larger studies. In light of our new findings, however, we recommend that genomic analyses, in particular, high-resolution microarrays as a first tier and ultimately whole-genome sequencing, be integrated into the standard of practice for diagnosis and clinical categorization of CP.

## Methods

### Participant selection

We recruited 161 individuals with a CP diagnosis made by a paediatric neurologist, developmental paediatrician or physiatrist to form a population-based cohort from nine rehabilitation centres from the Canadian provinces of Alberta, Quebec and Ontario; 293 parents were also collected. Informed consent was obtained from parents and the study was approved by the Research Ethics Boards at Holland Bloorview Kids Rehabilitation Hospital, the University of Alberta and McGill University. Detailed standardized information regarding the child's CP phenotypic profile including subtype, motor severity, medical co-morbidities, prenatal and perinatal factors were recorded.

### Microarray genotyping and quality control procedures

DNA was extracted from saliva samples obtained from each proband and their biological parents. These samples were genotyped on the Illumina HumanOmni2.5-8 (San Diego, CA, USA) at The Centre for Applied Genomics in Toronto using established protocols^34^,^35^. Samples were required to have a minimum call rate of 0.95. The s.d. for the LRR (log R ratio) and BAF (B allele frequency) for an individual sample were required to be within the mean±three times the s.d. for the entire cohort for each of these criteria. Any sample outside this range was removed from further analysis. CNV calls were made using four different CNV detection algorithms: iPattern^36^, PennCNV^37^, QuantiSNP^38^ and CNVPartition^36^. CNVs on autosomes required calling by at least two algorithms with one being either iPattern or PennCNV. CNV calls made on the X chromosome were only identified using iPattern and PennCNV and both algorithms were required to generate a stringent call. Very large CNVs were sometimes fragmented on account of both technical limitations of the array and the saliva samples used. As a result, all large CNVs were manually inspected and, if found to be fragmented, the calls were merged and sizes confirmed by examining the probe intensities and allele frequencies in the region (Supplementary Fig. 1). Parent–child relationships were confirmed using PLINK^39^. All relevant microarray data have been deposited in the Gene Expression Omnibus and can be accessed using Gene Expression Omnibus accession number GSE70374.

### Ancestry determination

The ancestry of the 147 cases successfully genotyped on the HumanOmni2.5-8 were determined using HapMap3 samples (CEU, TSI, YRI, JPT, CHD and CHB) genotyped on Genome-Wide Human SNP Array 6.0 as the reference set (Supplementary Data set 3). Single-nucleotide polymorphism (SNP) genotypes for both the HapMap and case samples were extracted and formatted for analysis using PLINK v1.90b2. After excluding the SNPs on the sex chromosomes, ambiguous SNPs, and those overlapping the major histocompatibility complex region, SNPs were filtered based on their minimum allele frequency (MAF) using the PLINK toolkit^39^. For each of the sets, all SNPs that had a genotyping rate <95% or where the MAF <5% were excluded. The SNPs common to the two platforms were extracted and the two data sets were combined. This time, all SNPs with a genotyping rate <95% or where the MAF <5% in the combined data set were removed. Linkage disequilibrium-based pruning of the autosomal SNPs with parameters 50 (window size), 5 (step) and 0.25 (*r*^2^ threshold) yielded 96,023 SNPs for the analysis. Population stratification and outlier detection were performed by multidimensional scaling analysis as implemented in PLINK. The top two principal components were then plotted using a custom R script (Supplementary Fig. 2).

### Rare variant detection

Rare variants were identified as those present in <0.1% of our population controls using <50% reciprocal overlap^40^. They were required to overlap (>75%) copy number stable regions of the genome^41^, be called by at least five successive probes, and exceed 10 kb in size. Our primary control data sets included 2,988 population control samples obtained from the KORA (Cooperative Research in the Region of Augsburg)^42^ and the COGEND (Collaborative Genetic Study of Nicotine Dependence)^43^, which were genotyped using the Illumina Human OMNI 2.5M-Quad microarray. We subsequently compared our CNV calls with those obtained from other population control individuals to further refine our list of rare variants. We utilized additional population control samples including 1,234 from the Ontario Heart Research Institute^44^ and 1,123 from the POPGEN^45^ (both cohorts were run on the Affymetrix 6.0 microarray); 1,769 controls from the SAGE consortium^46^, 433 controls from the Ontario Family Colorectal Cancer Registry^47^ and 2,566 from the Health, Aging and Body Composition study^48^ (from the Illumina 1M microarray); and 873 from the Ontario Population Genetics Platform^49^ (from the CytoScan HD).

### Validation of CNV findings

CNVs of potential clinical relevance (Table 3; Supplementary Table 2) were confirmed in patient and parental samples (where available) using a SYBR Green-based real-time quantitative PCR assay. Primer3 software v. 0.4.0 ( http://bioinfo.ut.ee/primer3-0.4.0/) was used to generate primer sequences that produce a PCR product of 90–140 bp. We also designed control primers to amplify a region of the *FOXP2* locus. NA10851 and NA15510 were used as our male and female controls and each experiment was performed in triplicate. Primer sequences used to amplify candidate regions can be found in Supplementary Table 3.

### Control sample permissions

We obtained the KORA, COGEND and Health ABC (HABC) control cohorts along with permission for use, from the database of Genotypes and Phenotypes found at http://www-ncbi-nlm-nih-gov.myaccess.library.utoronto.ca/gap through accession numbers phs000169.v1.p1 (Whole Genome Association Study of Visceral Adiposity in the HABC Study), phs000303.v1.p1 (Genetic Epidemiology of Refractive Error in the KORA Study) and phs000404.v1.p1 (COGEND; The Genetic Architecture of Smoking and Smoking Cessation). The Division of Aging Biology and the Division of Geriatrics and Clinical Gerontology, National Institute on Aging provided support for the ‘Center for Inherited Disease Research Visceral Adiposity Study'. HABC Study Investigators provided study coordination and assistance with phenotype harmonization and genotype cleaning. The National Eye Institute provided support for the KORA data set that was obtained from the NEI Refractive Error Collaboration Database. Genotyping of the COGEND samples was conducted at the Center for Inherited Disease Research and support was provided by 1 X01 HG005274-01. The Gene Environment Association Studies (GENEVA) Coordinating Center (U01HG004446) provided study coordination and assistance with genotype cleaning for these samples. The COGEND (P01 CA089392) and the University of Wisconsin Transdisciplinary Tobacco Use Research Center (P50 DA019706 and P50 CA084724) provided the support for sample collection for the COGEND samples and data sets. The contents of this article are solely the responsibility of the authors and do not necessarily represent the official views of the NIH.

## Additional information

**Accession codes:** All relevant microarray data has been deposited in the Gene Expression Omnibus (GEO) and can be accessed using GEO accession number GSE70374.

**How to cite this article:** Oskoui, M. *et al*. Clinically relevant copy number variations detected in cerebral palsy. *Nat. Commun.* 6:7949 doi: 10.1038/ncomms8949 (2015).

## Supplementary Material

Supplementary Information Supplementary Figure 1-2 and Supplementarty Tables 1-3

Supplementary Dataset 1 Rare microarray calls. This dataset contains the 412 rare CNV calls detected following the filtering procedure detailed in the manuscript.

Supplementary Dataset 2 Clinical Details. This dataset contains detailed clinical information for each of the probands discussed in the manuscript.

Supplementary Dataset 3 Ancestries of Cases. This dataset details the ancestry of each proband (as determined using their genotype) and provides the principle components from which we made the determination.

## Figures and Tables

**Table 1:** *De novo* CNV rates in related neurodevelopmental conditions.

**Cohort**	***De novo*** **CNV rate (%)**	**Study**	
Attention-deficit hyperactivity disorder	1.7–4.8	Lionel *et al*.^50^; Elia *et al*.^51^	
Autism	4.7–7.1	Sebat *et al*.^52^; Pinto *et al*.^53^	
Bipolar disorder	3.3–4.3	Malhotra *et al*.^54^; Noor *et al*.^55^	
Cerebral palsy	7.0	This study	
Epilepsy	5.0	Olson *et al*.^56^	
Severe intellectual disability	16.0–23.0	Gilissen *et al*.^57^; Qiao *et al*.^58^	
Schizophrenia	4.5–5.1	Malhotra *et al*.^55^; Kirov *et al*.^59^	
General population	0.9–1.4	Malhotra *et al*.^55^; Sanders *et al*.^60^	

**Table 2:** Clinical characteristics of CP probands.

*Patient characteristics*	N=*147*
Age in years (range)	7.86±3.15 (4–16)
Gender	
Male	81 (55%)
Female	66 (45%)
	
*Phenotypic characteristics*	
CP Subtype	
Spastic hemiplegia	55 (37.4%)
Spastic diplegia	34 (23.1%)
Spastic triplegia	6 (4.1%)
Spastic quadriplegia	31 (21.1%)
Ataxic CP	4 (2.7%)
Dyskinetic CP	12 (8.2%)
Unknown	5 (3.4%)
GMFCS	
I	59 (40.1%)
II	28 (19.0%)
III	13 (8.8%)
IV	19 (12.9%)
V	15 (10.2%)
Unknown	13 (8.8%)
Cortical visual impairment*	17.8%
Sensorineural auditory impairment†	10.9%
Communication difficulties‡	70.9%
Cognitive impairment§	42.5%
Feeding difficulties||	11.1%
Neonatal seizures¶	18.3%
Epilepsy#	15.5%

CP, Cerebral palsy; GMFCS, Gross Motor Function Classification System

^*^Cortical visual impairment status was available for 126 individuals.

^†^Sensorineural auditory impairment status was available for 128 individuals.

^‡^Status regarding communication difficulties was available for 134 individuals.

^§^The presence of cognitive impairment could be examined in 87 individuals.

^||^Gavage feeding and/or feeding by gastrostomy could be determined in 144 subjects.

^¶^We could evaluate the presence or absence of convulsions within the first 72 h for 120 individuals.

^#^The presence or absence of seizures in the last 12 months could be determined in 142 subjects.

**Table 3:**
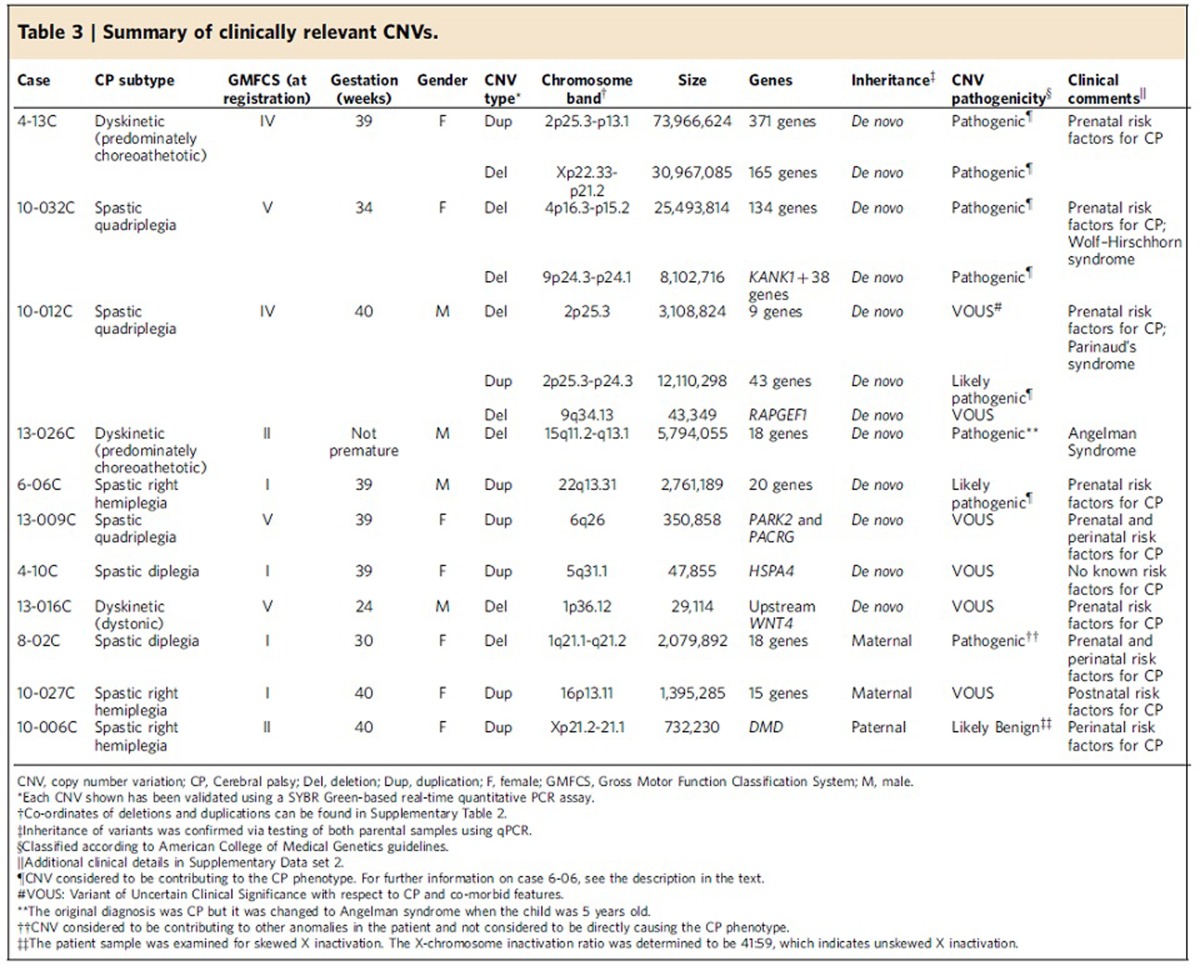
Summary of clinically relevant CNVs.

## References

[b1] Oskoui M., Coutinho F., Dykeman J., Jette N. & Pringsheim T. An update on the prevalence of cerebral palsy: a systematic review and meta-analysis. Dev. Med. Child Neurol. 55, 509–519 (2013). 23346889 10.1111/dmcn.12080

[b2] Himmelmann K., Ahlin K., Jacobsson B., Cans C. & Thorsen P. Risk factors for cerebral palsy in children born at term. Acta Obstet. Gynecol. Scand. 90, 1070–1081 (2011). 21682697 10.1111/j.1600-0412.2011.01217.x

[b3] McIntyre S. . A systematic review of risk factors for cerebral palsy in children born at term in developed countries. Dev. Med. Child Neurol. 55, 499–508 (2013). 23181910 10.1111/dmcn.12017

[b4] Beaino G. . Predictors of cerebral palsy in very preterm infants: the EPIPAGE prospective population-based cohort study. Dev. Med. Child Neurol. 52, e119–e125 (2010). 20163431 10.1111/j.1469-8749.2010.03612.x

[b5] Ellenberg J. H. & Nelson K. B. The association of cerebral palsy with birth asphyxia: a definitional quagmire. Dev. Med. Child Neurol. 55, 210–216 (2013). 23121164 10.1111/dmcn.12016

[b6] Rosenbaum P. . A report: the definition and classification of cerebral palsy April 2006. Dev. Med. Child Neurol. Suppl. 109, 8–14 (2007). 17370477

[b7] Wood E. & Rosenbaum P. The gross motor function classification system for cerebral palsy: a study of reliability and stability over time. Dev. Med. Child Neurol. 42, 292–296 (2000). 10855648 10.1017/s0012162200000529

[b8] Hemminki K., Li X., Sundquist K. & Sundquist J. High familial risks for cerebral palsy implicate partial heritable aetiology. Paediatr. Perinat. Epidemiol. 21, 235–241 (2007). 17439532 10.1111/j.1365-3016.2007.00798.x

[b9] Amor D. J., Craig J. E., Delatycki M. B. & Reddihough D. Genetic factors in athetoid cerebral palsy. J. Child Neurol. 16, 793–797 (2001). 11732763 10.1177/08830738010160110301

[b10] Lynex C. N. . Homozygosity for a missense mutation in the 67 kDa isoform of glutamate decarboxylase in a family with autosomal recessive spastic cerebral palsy: parallels with Stiff-Person Syndrome and other movement disorders. BMC Neurol. 4, 20 (2004). 15571623 10.1186/1471-2377-4-20 PMC544830

[b11] Lundy C., Lumsden D. & Fairhurst C. Treating complex movement disorders in children with cerebral palsy. Ulster Med. J. 78, 157–163 (2009). 19907680 PMC2773587

[b12] Lerer I. . Deletion of the ANKRD15 gene at 9p24.3 causes parent-of-origin-dependent inheritance of familial cerebral palsy. Hum. Mol. Genet. 14, 3911–3920 (2005). 16301218 10.1093/hmg/ddi415

[b13] Kakinuma N., Zhu Y., Wang Y., Roy B. C. & Kiyama R. Kank proteins: structure, functions and diseases. Cell. Mol. Life Sci. 66, 2651–2659 (2009). 19554261 10.1007/s00018-009-0038-y PMC11115667

[b14] Moreno-De-Luca A. . Adaptor protein complex-4 (AP-4) deficiency causes a novel autosomal recessive cerebral palsy syndrome with microcephaly and intellectual disability. J. Med. Genet. 48, 141–144 (2011). 20972249 10.1136/jmg.2010.082263 PMC3150730

[b15] Moreno-De-Luca A., Ledbetter D. H. & Martin C. L. Genetic [corrected] insights into the causes and classification of [corrected] cerebral palsies. Lancet Neurol. 11, 283–292 (2012). 22261432 10.1016/S1474-4422(11)70287-3 PMC3296129

[b16] Matsuda S. & Yuzaki M. Polarized sorting of AMPA receptors to the somatodendritic domain is regulated by adaptor protein AP-4. Neurosci. Res. 65, 1–5 (2009). 19481121 10.1016/j.neures.2009.05.007

[b17] Ashwal S. . Practice parameter: diagnostic assessment of the child with cerebral palsy: report of the Quality Standards Subcommittee of the American Academy of Neurology and the Practice Committee of the Child Neurology Society. Neurology 62, 851–863 (2004). 15037681 10.1212/01.wnl.0000117981.35364.1b

[b18] Shevell M., Miller S. P., Scherer S. W., Yager J. Y. & Fehlings M. G. The Cerebral Palsy Demonstration Project: a multidimensional research approach to cerebral palsy. Semin. Pediatr. Neurol. 18, 31–39 (2011). 21575839 10.1016/j.spen.2011.02.004

[b19] Gazzellone M. J. . Copy number variation in Han Chinese individuals with autism spectrum disorder. J. Neurodev. Disord. 6, 34 (2014). 25170348 10.1186/1866-1955-6-34 PMC4147384

[b20] MacDonald J. R., Ziman R., Yuen R. K., Feuk L. & Scherer S. W. The Database of Genomic Variants: a curated collection of structural variation in the human genome. Nucleic Acids Res. 42, D986–D992 (2014). 24174537 10.1093/nar/gkt958 PMC3965079

[b21] de Leeuw N. . Diagnostic interpretation of array data using public databases and internet sources. Hum. Mutat. 33, 930–940 (2012). 10.1002/humu.22049 PMC5027376 26285306

[b22] Miller D. T. . Consensus statement: chromosomal microarray is a first-tier clinical diagnostic test for individuals with developmental disabilities or congenital anomalies. Am. J. Hum. Genet. 86, 749–764 (2010). 20466091 10.1016/j.ajhg.2010.04.006 PMC2869000

[b23] Brunetti-Pierri N. . Recurrent reciprocal 1q21.1 deletions and duplications associated with microcephaly or macrocephaly and developmental and behavioral abnormalities. Nat. Genet. 40, 1466–1471 (2008). 19029900 10.1038/ng.279 PMC2680128

[b24] Lee C. & Scherer S. W. The clinical context of copy number variation in the human genome. Expert Rev. Mol. Med. 12, e8 (2010). 20211047 10.1017/S1462399410001390

[b25] Ramalingam A. . 16p13.11 duplication is a risk factor for a wide spectrum of neuropsychiatric disorders. J. Hum. Genet. 56, 541–544 (2011). 21614007 10.1038/jhg.2011.42

[b26] West A. B., Lockhart P. J., O'Farell C. & Farrer M. J. Identification of a novel gene linked to parkin via a bi-directional promoter. J. Mol. Biol. 326, 11–19 (2003). 12547187 10.1016/s0022-2836(02)01376-1

[b27] Pankratz N. . Parkin dosage mutations have greater pathogenicity in familial PD than simple sequence mutations. Neurology 73, 279–286 (2009). 19636047 10.1212/WNL.0b013e3181af7a33 PMC2715211

[b28] Bueter W., Dammann O. & Leviton A. Endoplasmic reticulum stress, inflammation, and perinatal brain damage. Pediatr. Res. 66, 487–494 (2009). 19668101 10.1203/PDR.0b013e3181baa083 PMC2776050

[b29] Kadotani T. . A chomosomal study on 100 cases of cerebral palsy. Int. J. Hum. Genet. 1, 109–112 (2001).

[b30] Segel R. . Copy number variations in cryptogenic cerebral palsy. Neurology 84, 1660–1668 (2015). 25817843 10.1212/WNL.0000000000001494

[b31] McMichael G. . Rare copy number variation in cerebral palsy. Eur. J. Hum. Genet. 22, 40–45 (2014). 23695280 10.1038/ejhg.2013.93 PMC3865415

[b32] Parolin Schnekenberg R. . De novo point mutations in patients diagnosed with ataxic cerebral palsy. Brain 138, 1817–1832 (2015). 25981959 10.1093/brain/awv117 PMC4572487

[b33] McMichael G. . Whole-exome sequencing points to considerable genetic heterogeneity of cerebral palsy. Mol. Psychiatry 20, 176–182 (2015). 25666757 10.1038/mp.2014.189

[b34] Scherer S. W. . Challenges and standards in integrating surveys of structural variation. Nat. Genet. 39, S7–15 (2007). 17597783 10.1038/ng2093 PMC2698291

[b35] Pinto D., Marshall C., Feuk L. & Scherer S. W. Copy-number variation in control population cohorts. Hum. Mol. Genet. 16, R168–R173 (2007). 17911159 10.1093/hmg/ddm241

[b36] Pinto D. . Comprehensive assessment of array-based platforms and calling algorithms for detection of copy number variants. Nat. Biotechnol. 29, 512–520 (2011). 21552272 10.1038/nbt.1852 PMC3270583

[b37] Wang K. . PennCNV: an integrated hidden Markov model designed for high-resolution copy number variation detection in whole-genome SNP genotyping data. Genome Res. 17, 1665–1674 (2007). 17921354 10.1101/gr.6861907 PMC2045149

[b38] Colella S. . QuantiSNP: an Objective Bayes Hidden-Markov Model to detect and accurately map copy number variation using SNP genotyping data. Nucleic Acids Res. 35, 2013–2025 (2007). 17341461 10.1093/nar/gkm076 PMC1874617

[b39] Purcell S. . PLINK: a tool set for whole-genome association and population-based linkage analyses. Am. J. Hum. Genet. 81, 559–575 (2007). 17701901 10.1086/519795 PMC1950838

[b40] Pinto D. . Functional impact of global rare copy number variation in autism spectrum disorders. Nature 466, 368–372 (2010). 20531469 10.1038/nature09146 PMC3021798

[b41] Zarrei M., MacDonald J. R., Merico D. & Scherer S. W. A copy number variation map of the human genome. Nat. Rev. Genet. 16, 172–183 (2015). 25645873 10.1038/nrg3871

[b42] Verhoeven V. J. . Genome-wide meta-analyses of multiancestry cohorts identify multiple new susceptibility loci for refractive error and myopia. Nat. Genet. 45, 314–318 (2013). 23396134 10.1038/ng.2554 PMC3740568

[b43] Bierut L. J. . Novel genes identified in a high-density genome wide association study for nicotine dependence. Hum. Mol. Genet. 16, 24–35 (2007). 17158188 10.1093/hmg/ddl441 PMC2278047

[b44] Stewart A. F. . Kinesin family member 6 variant Trp719Arg does not associate with angiographically defined coronary artery disease in the Ottawa Heart Genomics Study. J. Am. Coll. Cardiol. 53, 1471–1472 (2009). 19371834 10.1016/j.jacc.2008.12.051

[b45] Krawczak M. . PopGen: population-based recruitment of patients and controls for the analysis of complex genotype-phenotype relationships. Community. Genet. 9, 55–61 (2006). 16490960 10.1159/000090694

[b46] Bierut L. J. . A genome-wide association study of alcohol dependence. Proc. Natl Acad. Sci. USA 107, 5082–5087 (2010). 20202923 10.1073/pnas.0911109107 PMC2841942

[b47] Cotterchio M. . Red meat intake, doneness, polymorphisms in genes that encode carcinogen-metabolizing enzymes, and colorectal cancer risk. Cancer Epidemiol. Biomarkers Prev. 17, 3098–3107 (2008). 18990750 10.1158/1055-9965.EPI-08-0341 PMC2751598

[b48] Coviello A. D. . A genome-wide association meta-analysis of circulating sex hormone-binding globulin reveals multiple Loci implicated in sex steroid hormone regulation. PLoS Genet. 8, e1002805 (2012). 22829776 10.1371/journal.pgen.1002805 PMC3400553

[b49] Uddin M. . A high-resolution copy-number variation resource for clinical and population genetics. Genet. Med. doi:10.1038/gim.2014.178 (2014). PMC4752593 25503493

[b50] Lionel A. C. . Rare copy number variation discovery and cross-disorder comparisons identify risk genes for ADHD. Sci. Transl. Med. 3, 95ra75 (2011). 10.1126/scitranslmed.3002464 21832240

[b51] Elia J. . Genome-wide copy number variation study associates metabotropic glutamate receptor gene networks with attention deficit hyperactivity disorder. Nat. Genet. 44, 78–84 (2012). 22138692 10.1038/ng.1013 PMC4310555

[b52] Sebat J. . Strong association of de novo copy number mutations with autism. Science 316, 445–449 (2007). 17363630 10.1126/science.1138659 PMC2993504

[b53] Pinto D. . Convergence of genes and cellular pathways dysregulated in autism spectrum disorders. Am. J. Hum. Genet. 94, 677–694 (2014). 24768552 10.1016/j.ajhg.2014.03.018 PMC4067558

[b54] Noor A. . Copy number variant study of bipolar disorder in Canadian and UK populations implicates synaptic genes. Am. J. Med. Genet. B Neuropsychiatr. Genet. 165B, 303–313 (2014). 24700553 10.1002/ajmg.b.32232

[b55] Malhotra D. . High frequencies of de novo CNVs in bipolar disorder and schizophrenia. Neuron 72, 951–963 (2011). 22196331 10.1016/j.neuron.2011.11.007 PMC3921625

[b56] Olson H. . Copy number variation plays an important role in clinical epilepsy. Ann. Neurol. 75, 943–958 (2014). 24811917 10.1002/ana.24178 PMC4487364

[b57] Gilissen C. . Genome sequencing identifies major causes of severe intellectual disability. Nature 511, 344–347 (2014). 24896178 10.1038/nature13394

[b58] Qiao Y. . Copy number variants (CNVs) analysis in a deeply phenotyped cohort of individuals with intellectual disability (ID). BMC Med. Genet. 15, 82 (2014). 25030379 10.1186/1471-2350-15-82 PMC4107469

[b59] Kirov G. . *De novo* CNV analysis implicates specific abnormalities of postsynaptic signalling complexes in the pathogenesis of schizophrenia. Mol. Psychiatry 17, 142–153 (2012). 22083728 10.1038/mp.2011.154 PMC3603134

[b60] Sanders S. J. . Multiple recurrent de novo CNVs, including duplications of the 7q11.23 Williams syndrome region, are strongly associated with autism. Neuron 70, 863–885 (2011). 21658581 10.1016/j.neuron.2011.05.002 PMC3939065

