# Supplementary material for: Clinically relevant copy number variations detected in cerebral palsy
Source: Nat Commun. 2015 Aug 3;6:7949. doi: 10.1038/ncomms8949 (PMC4532872; doi:10.1038/ncomms8949)
Supplement: Supplementary Information — Supplementary Figure 1-2 and Supplementarty Tables 1-3 [file ncomms8949-s1.pdf]

Supplementary Information for "Clinically Relevant Copy Number Variations Detected In Cerebral Palsy"

Maryam Oskoui[1,*], Matthew J. Gazzellone[2,3,*], Bhooma Thiruvahindrapuram[2,3], Mehdi Zarrei[2,3], John Andersen[4], John Wei[2,3], Zhuozhi Wang[2,3], Richard F. Wintle[2,3], Christian R. Marshall[2,3,5], Ronald D. Cohn[3,6,7,8], Rosanna Weksberg[3,6,9], Dimitri J. Stavropoulos[10], Darcy Fehlings[11], Michael I. Shevell[1], Stephen W. Scherer[2,3,12]

[1]Departments of Pediatrics and Neurology/Neurosurgery, McGill University, Montreal, Quebec, Canada, H3H 1P3

[2]The Centre for Applied Genomics, The Hospital for Sick Children, Toronto, Ontario, Canada, M5G 0A4

[3]Program in Genetics and Genome Biology, The Hospital for Sick Children, Toronto, Ontario, Canada, M5G 0A4

[4]Department of Pediatrics, University of Alberta, Edmonton, Alberta, Canada. Glenrose Rehabilitation Hospital, Edmonton, Alberta, Canada, T5G 0B7

[5]Genome Diagnostics, Department of Pediatric Laboratory Medicine, The Hospital for Sick Children, Toronto, Ontario, Canada, M5G 1X8

[6]Department of Paediatrics, University of Toronto, Toronto, Ontario, Canada, M5G 1X8

[7]Centre for Genetic Medicine, The Hospital for Sick Children, Toronto, Ontario, Canada, M5G 1X8

[8]Division of Clinical and Metabolic Genetics, The Hospital for Sick Children, Toronto, Ontario, Canada, M5G 1X8

[9]Institute of Medical Science, University of Toronto, Toronto, Ontario, Canada, M5G 1X8

[10]Cytogenetics Laboratory, Department of Pediatric Laboratory Medicine, Hospital for Sick Children, Toronto, Ontario, Canada. Department of Laboratory Medicine and Pathobiology Hospital for Sick Children, Toronto, Ontario, Canada, M5G 1X8

[11]Holland Bloorview Kids Rehabilitation Hospital, Department of Paediatrics, University of Toronto, Toronto, Ontario, Canada, M4G 1R8

[12]Department of Molecular Genetics and McLaughlin Centre, University of Toronto, Toronto, Ontario, Canada, M5S 1A8.

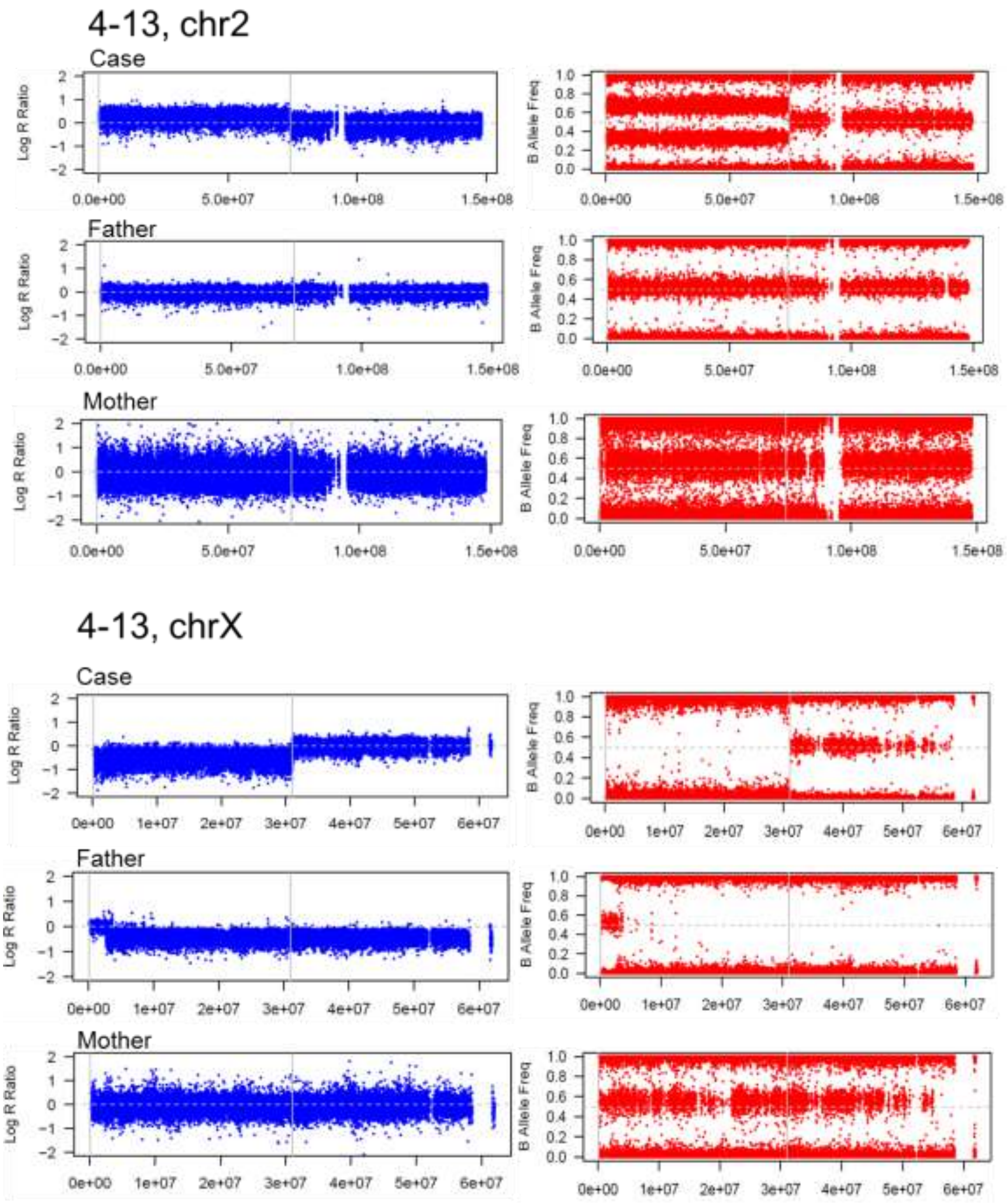

Supplementary Figure 1 | Probe Intensities and Allele Frequencies for Large *De novo* CNVs

These panels illustrate the probe intensities and allele frequencies at the regions containing large *de novo* CNVs (>2 Mb) for cases. We also show the same region for parents. The left panel shows the probe intensities and the right illustrates the allele frequencies. The sample ID and chromosome containing the aberration are in the header for each trio according to Table 3.

10-032, chr4

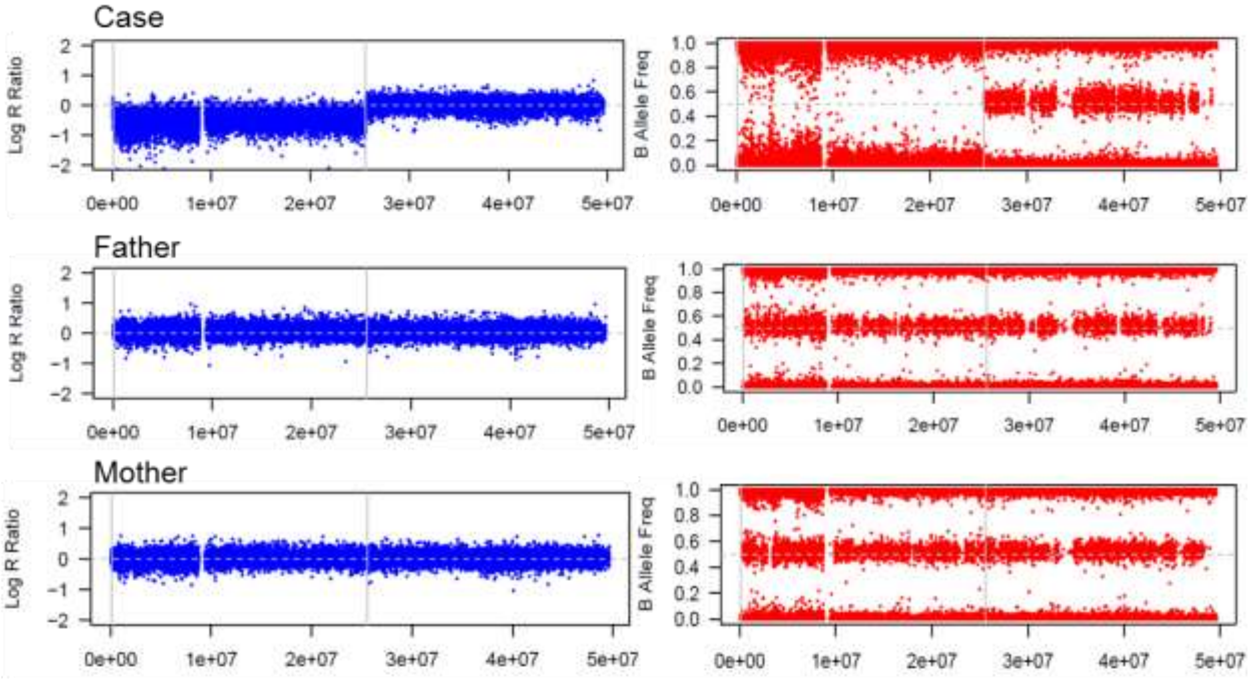

10-032, chr9

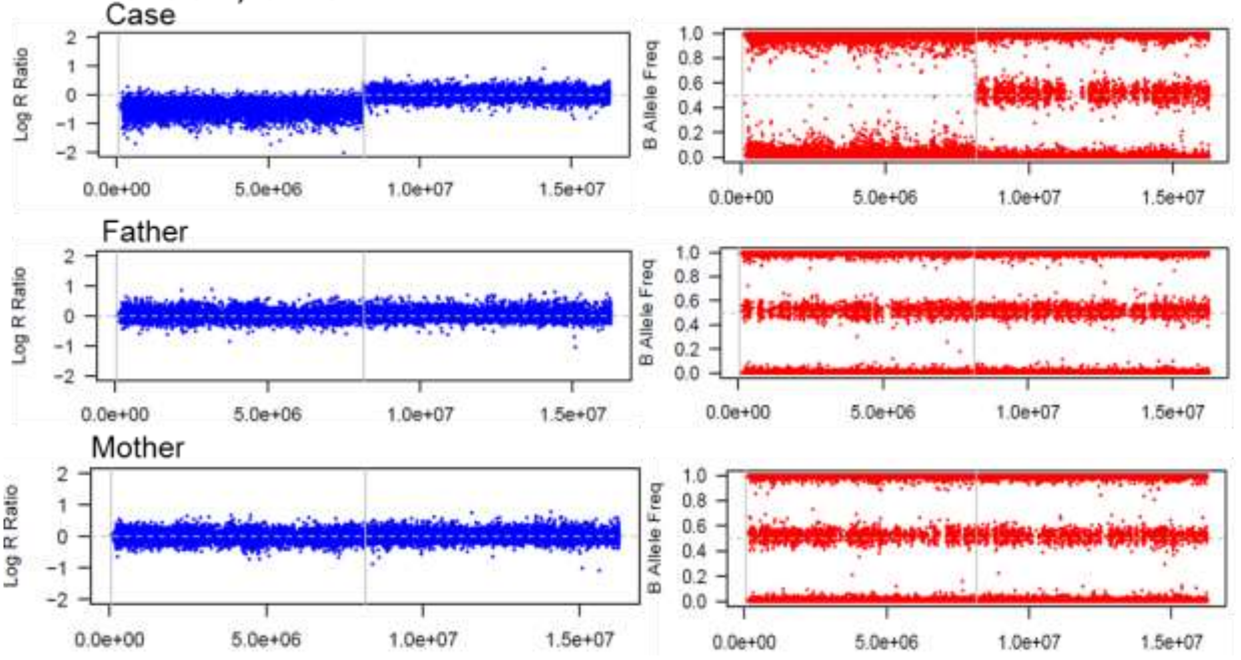

Supplementary Figure 1 | Continued

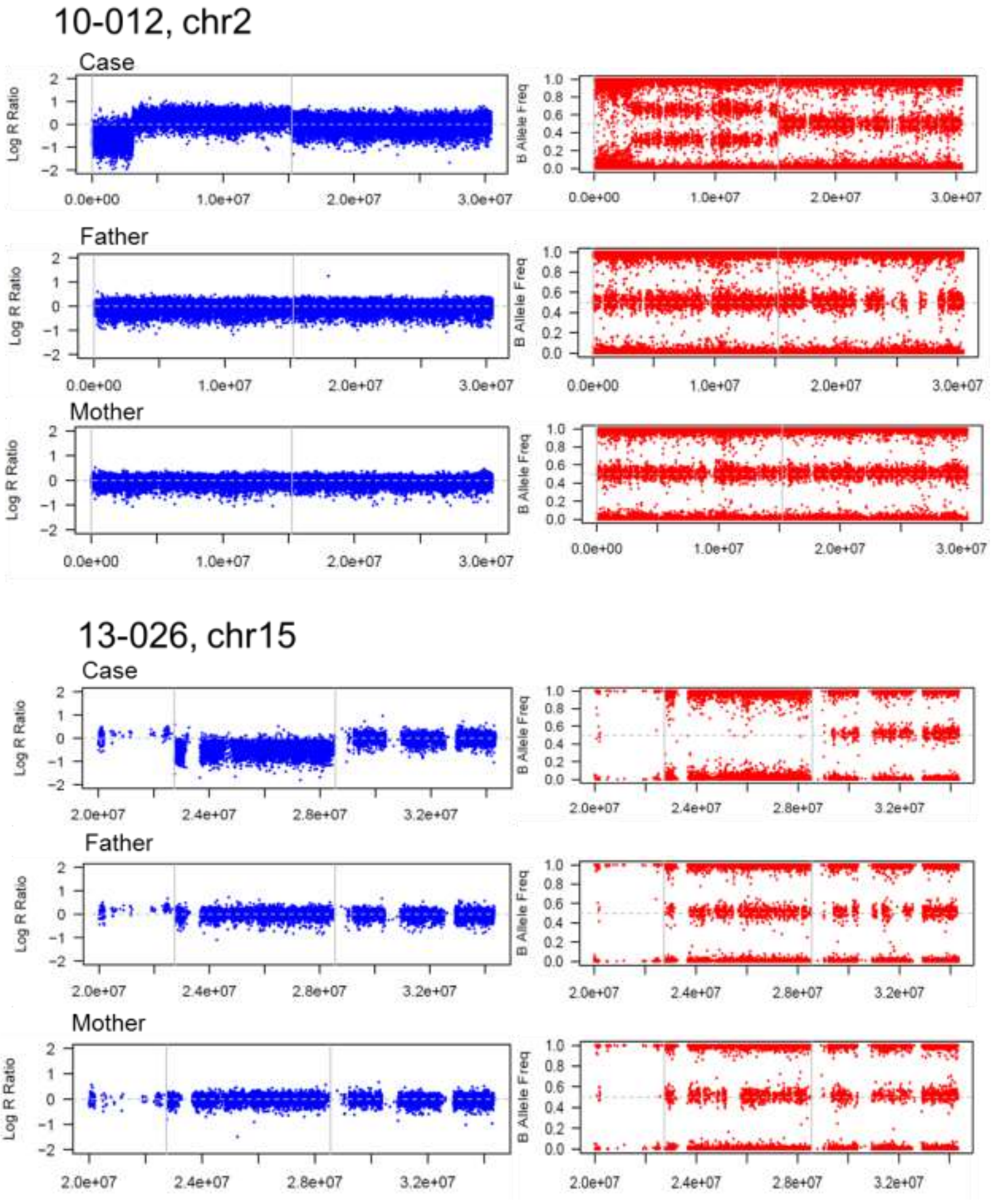

Supplementary Figure 1 | Continued

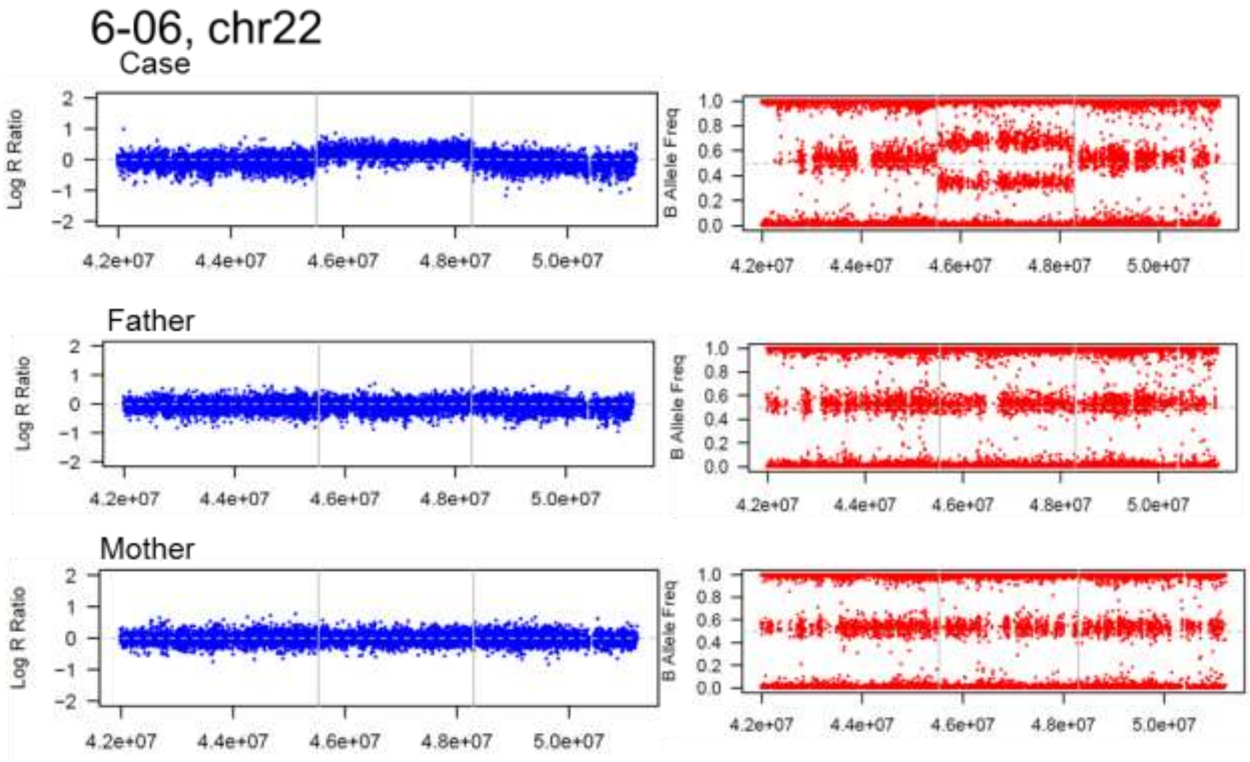

Supplementary Figure 1 | Continued

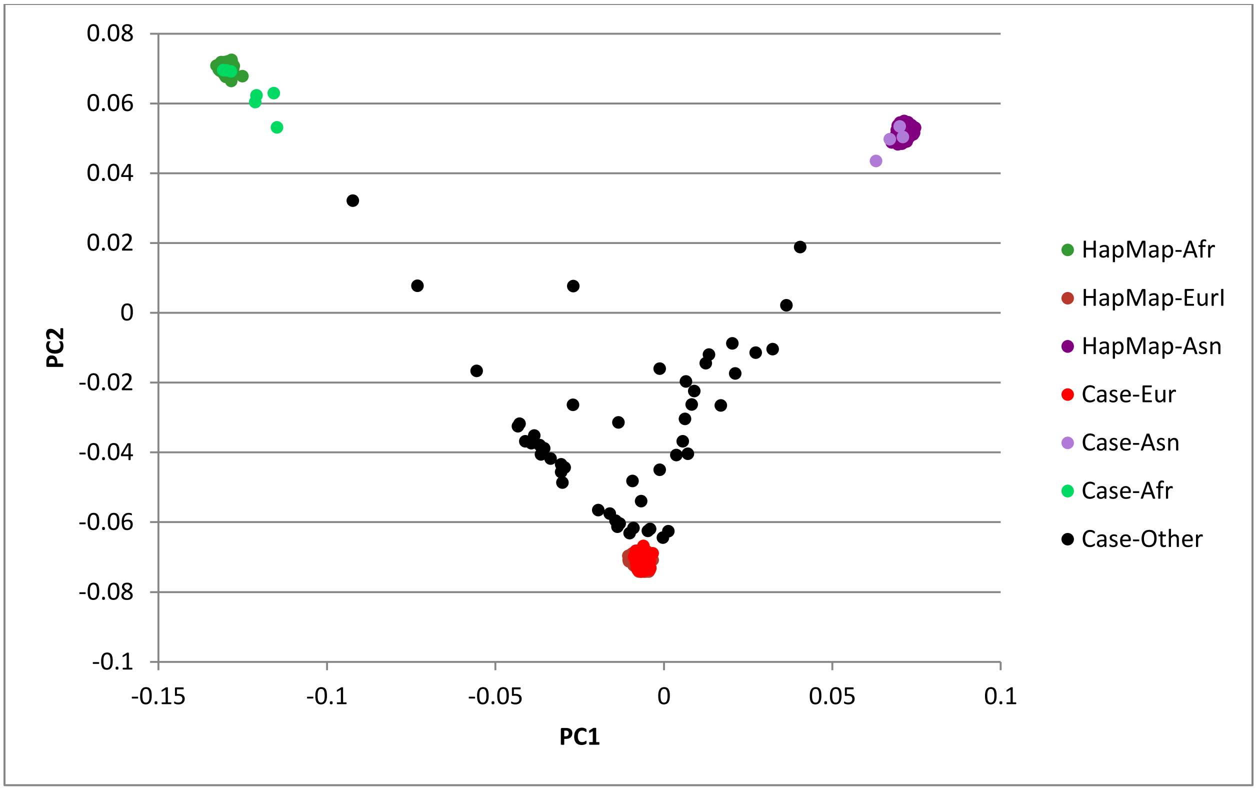

Supplementary Figure 2 | Ancestry Determination for CP Cases

The two principle components from the multidimensional scaling analysis are plotted above to illustrate the varied ancestries of the cases.

Supplementary Table 1: Summary Statistics of Unannotated Stringent CNVs larger than 10 kb

	**CP Probands**	**Parents**
Samples	147	282
Males/Females	81/66	134/148
#Stringent CNVs	3,277	6,087
Mean CNVs/sample ± SD[a]	22.29 ± 6.17	21.59 ± 5.32
Median	22	22
Mean CNV size (kb) ± SD[b]	145,034± 1,112,276.70	98,756± 169,746.3
Median CNV size (kb)	40,055	37,915
%Gain/%Loss	41.7/58.3	39.9/60.1
#CNVs > 1Mb (%)	32 (0.98%)	41 (0.67%)
#CNVs 100kb-1Mb (%)	900 (27.46%)	1,543 (25.35%)
#CNVs 10 kb-100kb (%)	2,345 (71.56%)	4,503 (73.98%)

[a] There is no significant difference between the mean number of CNV calls in CP probands and their parents (p=0.2219 using an unpaired two-tailed student's t test).

[b] There is no difference between the mean CNV size in CP probands and their parents (p=0.4942).

Supplementary Table 2: CNV Position for CNVs of Interest

Sample	chr	start (hg19)	End (hg19)	Size	CNV	Gender	Inheritance
4-13C	2	14,238	73,980,862	73,966,624	Gain	F	dn
4-13C	X	60,425	31,027,510	30,967,085	Loss	F	dn
10-032C	4	71,566	25,565,380	25,493,814	Loss	F	dn
10-032C	9	46,587	8,149,303	8,102,716	Loss	F	dn
10-012C	2	14,238	3,123,061	3,108,824	Loss	M	dn
10-012C	2	3,124,598	15,234,896	12,110,298	Gain	M	dn
10-012C	9	134,420,585	134,463,933	43,349	Loss	M	dn
13-026C	15	22,750,305	28,544,359	5,794,055	Loss	M	dn
6-06C	22	45,528,073	48,289,262	2,761,189	Gain	M	dn
13-009C	6	163,059,403	163,410,260	350,858	Gain	F	dn
4-10C	5	132,419,766	132,467,620	47,855	Gain	F	dn
13-016C	1	22,514,676	22,543,789	29,114	Loss	M	dn
8-02C	1	146,472,911	148,552,802	2,079,892	Loss	F	mat
10-027C	16	14,967,955	16,363,239	1,395,285	Gain	F	mat
10-006C	X	31,402,175	32,134,404	732,230	Gain	F	pat
8-03C	6	162,603,101	162,629,077	25,977	Loss	M	mat
3-07C	6	163,475,888	163,490,190	14,303	Loss	F	pat

dn=de novo; mat=maternal; pat=paternal

Supplementary Table 3: Primer Sequences for qPCR Validation

Targeted Locus	Size of targeted CNV (bp)	Forward Primer	Reverse Primer
FOXP2	CONTROL	TGCTAGAGGAGTGGGACAAGTA	GAAGCAGGACTCTAAGTGCAGA
2p25.3-p13.1	73,966,624	GGCATCTACTTTAGGCTGTGC	CTGAGCCAGTCTCTGATCCA
2p25.3-p13.1	73,966,624	CAAATCATCTGCTCACGATCTC	GTACAGCGTGTGGAACTAGCC
Xp22.33-p21.2	30,967,085	TGTCTCAACTGGAGTCTCTTGG	CAGTCATCTTGGAACCCAAAC
Xp22.33-p21.2	30,967,085	CATGCAGTGCACCATGGTTAT	ACAAATTGGTTGAGTTTCCCG
4p16.3-p15.2	25,493,814	TAATTGTGGTGCTTGGTCTCAC	GGTGGGATGCAAATTATTGAGT
4p16.3-p15.2	25,493,814	TCTCACTTCCTGTTTAGGTTAGACATT	GTGTCCTGTACTTTAGGTTGCATCT
9p24.3-p24.1	8,102,716	CAGATGAGGTATGCCAAGGAA	CTTCCACTAACAGTCACCACCA
9p24.3-p24.1	8,102,716	GACTACAGAATGCAGTGGGTGA	TTATGCCTGCACTACCAAACAG
2p25.3	3,108,824	GCATCCAACTCATCTCAGCA	CACTACAAGCTACAAAGGAGTTTACG
2p25.3-p24.3	12,110,298	GGCATCTACTTTAGGCTGTGC	CTGAGCCAGTCTCTGATCCA
2p25.3-p24.3	12,110,298	CAAATCATCTGCTCACGATCTC	GTACAGCGTGTGGAACTAGCC
9q34.13	43,349	GGAGAATTTCAGTTCACACAACC	GAAGTCCCTTCATTTCAATCCTT
15q11.2-q13.1	5,794,055	TGATCCTCTGTGATTGTGATGAG	GTTACCCTCGGAAATCCCTTAC
15q11.2-q13.1	5,794,055	ACCTAGAGATAAAGGTCTGAAGCAA	AACTACAGAATATGACGGTGGCTA
22q13.31	2,761,189	TATCCCTCCAACACAACTGGTA	GGGACTTGAAAGCAGATTAAGG
6q26	350,858	TTCCTTGTCATAATTCCTCCTTG	TCCCACCCAGCTTTGTCTAT
6q26	350,858	GAGCTTAAACAAATGCCCAGAC	GTGAAACCCTCAGAAACCAGAG
5q31.1	47,855	CCATCTTATCGCCTGCTTTC	AACATGATACCTTGACCCTTCTTC
5q31.1	47,855	CCTCAATGGGCAGTTGTGTA	ATGTGATTTGCCTACAGGTTCTC
1p36.12	29,114	TCAACTCATCTCTTGGGACAGA	TCTTTCTGCTAACACTCCTCACC
1p36.12	29,114	AGCTGTAAGTGCAGGAAGTGTG	TCAGATGAGGAATCAGGGTGT
1q21.1-q21.2	2,079,892	ACACATATCAGCCTCAAAGCAA	TTCTTGCCTCTAAGGATTCAGG
1q21.1-q21.2	2,079,892	GATATACTAAGTGGCCGCAGAAA	GTTGAATCTAAGGGTCCAGGAAT
16p13.11	1,395,285	GACTCTCACATAGGCACCAACA	ATAGACCTTCTTCATCCCAGCA
Xp21.2-21.1	732,230	TGATGATTCAACCCTCTTGGT	CGATCTCAAGAATTGCCTGTT
Xp21.2-21.1	732,230	TTGCTAGAGGTTGCTTCATTACC	AACCGGATGTGGAAGAGATTT
6q26	25,977	AACAGCCCAGTGAATGTTGA	GGCTTCTGTTACGGTGTGATTA
6q26	14,303	TCTATGCTCCAGTTCCAACAGA	TCTGCTCACAGACTTCCCATT
6q26	14,303	AGATCCTACCTGTCCTTCCACA	ATCACCTCAGTCTGTCATGGTTT

Supplementary Dataset 1 | Rare microarray calls (see online Supplementary_Dataset_1.xlsx)

This dataset contains the 412 rare CNV calls detected following the filtering procedure detailed in the manuscript.

Supplementary Dataset 2 | Clinical Details (see online Supplementary_Dataset_2.xlsx)

This dataset contains detailed clinical information for each of the probands discussed in the manuscript.

Supplementary Dataset 3 | Ancestries of Cases (see online Supplementary_Dataset_3.xlsx)

This dataset details the ancestry of each proband (as determined using their genotype) and provides the principle components from which we made the determination.
